# Constraints on Upward Migration of Hydraulic Fracturing Fluid and Brine

**DOI:** 10.1111/gwat.12095

**Published:** 2013-07-29

**Authors:** Samuel A Flewelling, Manu Sharma

**Affiliations:** 2 Gradient, 20 University RoadCambridge, MA 02138; msharma@gradientcorp.com

## Abstract

Recent increases in the use of hydraulic fracturing (HF) to aid extraction of oil and gas from black shales have raised concerns regarding potential environmental effects associated with predictions of upward migration of HF fluid and brine. Some recent studies have suggested that such upward migration can be large and that timescales for migration can be as short as a few years. In this article, we discuss the physical constraints on upward fluid migration from black shales (e.g., the Marcellus, Bakken, and Eagle Ford) to shallow aquifers, taking into account the potential changes to the subsurface brought about by HF. Our review of the literature indicates that HF affects a very limited portion of the entire thickness of the overlying bedrock and therefore, is unable to create direct hydraulic communication between black shales and shallow aquifers via induced fractures. As a result, upward migration of HF fluid and brine is controlled by preexisting hydraulic gradients and bedrock permeability. We show that in cases where there is an upward gradient, permeability is low, upward flow rates are low, and mean travel times are long (often >10^6^ years). Consequently, the recently proposed rapid upward migration of brine and HF fluid, predicted to occur as a result of increased HF activity, does not appear to be physically plausible. Unrealistically high estimates of upward flow are the result of invalid assumptions about HF and the hydrogeology of sedimentary basins.

## Introduction

The use of hydraulic fracturing (HF) in conjunction with the development of black shales has prompted questions regarding the potential for upward migration of HF fluid and brine through bedrock. A few recent studies have considered this possibility (Rozell and Reaven 2012; Myers 2012; Warner et al. 2012), however, none has provided a thorough discussion of the physical setting of black shales or the factors that control fluid migration at depth. These studies suggest that there is either a preexisting hydraulic connection between black shales and shallow groundwater or that HF may create such hydraulic connection and allow brine or HF fluid to migrate upward into shallow groundwater. Myers (2012) proposed that such migration could occur in less than 10 years; Rozell and Reaven (2012) predicted that, on average, over 200 m^3^ of HF fluid could leak into a shallow aquifer from any given deep gas well; (Warner et al. 2012) did not specify timescales for transport or volumetric fluxes, but they did suggest that hydraulic communication between black shales and shallow aquifers exists in parts of the Marcellus Shale region in Pennsylvania and that such areas may provide preferential pathways for HF fluid migration. These suggestions, regarding high flow rates and short travel times, contradict the body of literature on the hydrology of sedimentary basins.

In this article, we discuss the constraints on upward fluid migration from black shales to shallow aquifers. Our analysis applies to a number of black shales that are currently being targeted for oil and gas development, such as the Marcellus, the Barnett, the Bakken, the Niobrara, and the Eagle Ford. Not surprisingly, our discussion focuses on permeabilities, head gradients, and the relationships between the two, as these variables control the direction and magnitude of vertical fluxes. We show that in cases where upward head gradients exist, permeability is low, and therefore, vertical fluxes are low. Additionally, timescales for transport are long (often >10^6^ years). Hydraulic fracturing increases permeability at depth, however, it affects a much smaller thickness than that of the overlying bedrock and occurs over too short a timescale to affect natural vertical head gradients. After an HF stimulation, hydrocarbon extraction creates a low pressure zone that draws fluids toward the target formation, thereby eliminating any potential for upward flow. In sum, rapid upward migration of HF fluid or brine via bedrock would require the co-occurrence of upward head gradients and high bedrock permeabilities. As we discuss in this article, these two conditions are mutually exclusive, indicating that widespread and rapid upward migration of HF fluid and brine through bedrock is not physically plausible.

## Hydrogeological Setting of Black Shales Within Sedimentary Basins

**Figure 1:**
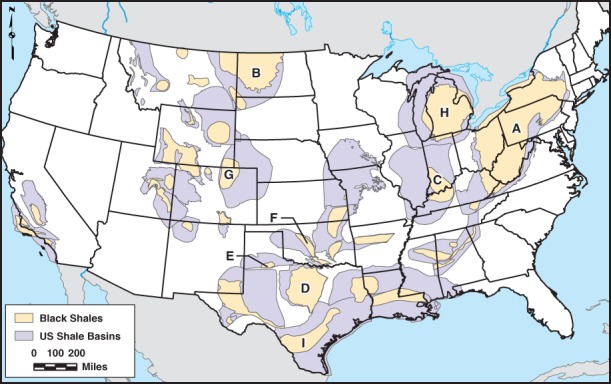
Location of major basins and black shales in the United States. Some of the black shales are labeled with letters, as follows, to provide some points of reference for later discussion: (A) Marcellus & Utica Shales, the Appalachian Basin; (B) Bakken Shale, the Williston Basin; (C) New Albany Shale, the Illinois Basin; (D) Barnett Shale, the Fort Worth Basin; (E) Bend Shale, the Palo Duro Basin; (F) Woodford Shale, the Anadarko Basin; (G) Niobrara Shale, the Denver Basin; (H) Antrim Shale, the Michigan Basin; and (I) Eagle Ford/Pearsall Shale, the Western Gulf Basin.

**Figure 2:**
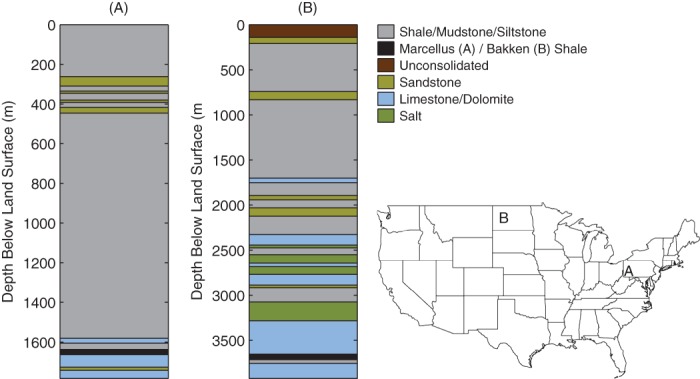
Dominant overburden stratigraphy above the Marcellus (A; after Ryder et al. 2012) and Bakken (B; after Sandberg 1962) Shales, located in the Appalachian and Williston Basins, respectively. Note that the vertical scale differs in the two stratigraphic columns. Inset map shows the approximate location of each stratigraphic column. Although the stratigraphy varies between the two basins, the overburden rocks above both black shales are primarily low permeability shales, siltstones, and mudstones. Note also that multiple low permeability salt beds overlie the Bakken Shale. More detailed information on stratigraphy in the Appalachian and Williston Basins is described by (Ryder et al. 2012) and Sandberg (1962), respectively.

Sedimentary basins occur around the globe, including many in the United States (Hunt 1990). The thickness of sediment in U.S. basins varies depending on their history of formation, uplift, and subsequent erosion; in some cases, sediment thicknesses in excess of 10 km accumulated during periods of deposition (e.g., in portions of the Appalachian Basin during the Permian—circa 300 to 250 million years ago; Garven et al. 1993; Rowan 2006). The locations of sedimentary basins in the United States containing black shales are shown in Figure 1. The overburden rocks above the targeted black shales are predominantly fine-grained (e.g., shale or mudstone; Figure 2) or mixtures of fine-grained and coarse-grained rocks (e.g., shaly sandstone; Sandberg 1962; Kiteley 1978; Baird and Dyman 1993; Ryder et al. 2008, 2009, 2012; Swezey 2008, 2009).

At all depths (beginning typically within 100 m of the surface), fluids are present in the sedimentary column, including fresh water, brine, oil, and natural gas (Bredehoeft 2003). Fluids may circulate to depths as great as 10 to 15 km or deeper (Nur and Walder 1990), i.e., throughout the entire vertical extent of sedimentary basins. Although fluids do circulate, flow in the deeper portions of basins tends to be very slow (Toth 1962, 1963), leading to basin-scale travel times that may be millions of years or longer (Kreitler 1989; Hogan et al. 2007). Brine is the dominant fluid (Hanor 1983), although oil and natural gas may also be present, trapped in isolated pockets or low permeability layers.

All sedimentary basins have layered structures, although sediment thickness and stratigraphy vary within and between basins (Miall 2008). This layered structure has a major influence on fluid migration, causing flow in high permeability layers to be generally parallel to the direction of bedding, while flow in low permeability layers is perpendicular to bedding (Freeze and Witherspoon 1967).

## Constraints on Permeability

Permeability in sedimentary basins is inherently anisotropic across a range of spatial scales (Desbarats 1987; Clennell et al. 1999), where horizontal permeability is often an order of magnitude or more greater than vertical permeability. The average or effective vertical permeability (*k*_eff_) for flow perpendicular to bedding is approximated as a harmonic mean (Kreitler 1989), where *L* and *k* are the thickness and permeability of strata, respectively, 
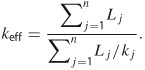
1

In the layered structure of sedimentary basins, *k*_eff_ will be dominated by the least permeable rock layer, even in cases where there is only a thin low-*k* layer. For example, if we assume permeability values for shale (10^−18^ m^2^) and sandstone (10^−15^ m^2^) (Freeze and Cherry 1979) and calculate *k*_eff_ from Equation 1 for a 1000 m thick rock column, of which 20 m are shale and 980 m are sandstone, *k*_eff_ is 5 × 10^−17^ m^2^. That is, *k*_eff_ is only 5% of the permeability of sandstone, even though sandstone comprises 98% of the hypothetical section. More commonly, stratigraphy above black shales is dominated by fine-grained rocks (e.g., shales and mudstones) and therefore, multiple, sometimes thick, low-*k* layers may limit vertical flow rates (Sandberg 1962; Baird and Dyman 1993; Ryder et al. 2008, 2009, 2012). Two examples of shale-dominated overburden are shown in Figure 2. Low *k*_eff_ makes intuitive sense because the rocks must have low permeability in order to have trapped buoyant fluids (i.e., oil and natural gas) over timescales of tens to hundreds of millions of years (Connolly et al., 1990a,1990b; Stueber and Walter 1991; Thornton and Wilson 2007).

### Causes of Low Permeability at Depth

The grain-size distribution is the dominant control on permeability, however, other factors are also important at depth, including effective stress, partial saturation, and cementation, often reducing permeability by orders of magnitude.

**Figure 3:**
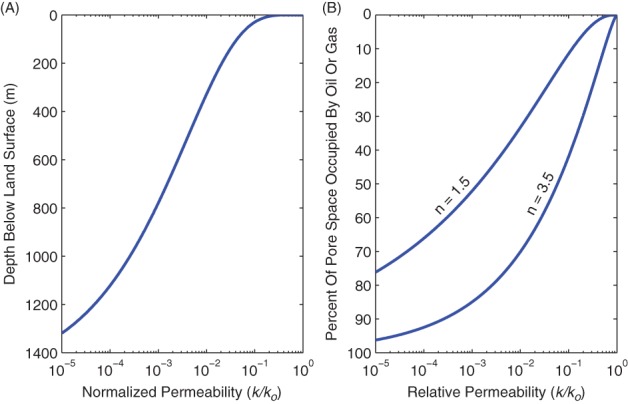
(A) Normalized permeability of Wilcox Shale from (Kwon et al. 2001), assuming that effective stress is the difference between lithostatic pressure and hydrostatic pressure. The bulk density of overburden and water were assumed to be 2300 kg m^−3^ and 1000 kg m^−3^, respectively. *k_0_* in this case would be the permeability at the land surface (i.e., when effective stress is zero); (B) relative permeability estimated from Equation 2. *k_0_* in this case would be the permeability for water-saturated rock. In gas-rich shales, pore space is predominantly occupied by gas and oil; therefore, the permeability to water is reduced by orders of magnitude.

Permeability is partly dependent on effective stress, which controls the amount of compaction and fracture apertures in a given rock layer. Both the void space and connectivity decrease as effective stress increases, thereby restricting flow and lowering permeability. (Kwon et al. 2001) provided a pressure-permeability relationship for the Wilcox Shale based on laboratory experiments, *k* = *k*_0_[1 − (*P*_e_/*P*_1_)^*m*^]^3^, where *k*_0_ is on the order of 10^−17^ m^2^, *P*_1_ is 19.3 (±1.6) MPa, *m* is 0.159 (±0.007), and *P*_e_ is the effective stress (*P*_e_ = *P*_c_ − *χP*_p_, where *P*_c_ is the overburden stress, *P*_p_ is fluid pore pressure, and *χ* is a constant that is approximately one for shales; Kwon et al. 2001). This relationship is plotted in Figure 3A. Note that the relationship of (Kwon et al. 2001) is for horizontal permeability (for flow parallel to bedding), which is typically higher than vertical permeability (for flow perpendicular to bedding). (Kwon et al. 2001) indicate that permeability decreases by 4 orders of magnitude as effective stress increases to 12 MPa (e.g., conditions that may be encountered at depths >1000 m).

The presence of multiple fluid phases (e.g., oil, natural gas, and water) in porous media also reduces permeability. One common relationship for relative permeability (*K*_r_) is given below, where *S* is saturation and *n* is a fitted parameter (Brooks and Corey 1964; van Genuchten 1980; Morel-Seytoux et al. 1996), 
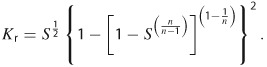
2

The relationship between *S* and *K*_r_ for water (from Equation 2) is depicted in Figure 3B for values of *n* ranging from 1.5 to 3.5 (for a broad range of grain-size distributions; Bohne et al. 1992). Permeability is sometimes described as being effectively zero if *S* drops below a critical value, below which the fluid exists as residual water bound to the porous matrix (Pallatt and Thornley 1990). Although permeability is never truly zero, migration of bound water may occur via non-Darcian mechanisms, such as diffusion—a very slow process. Low water saturation is common in source rocks (e.g., black shales) and reservoir rocks, and thus, the permeability of these layers to water is very low. In the Marcellus Shale, for example, natural gas almost fully occupies the available pore space, meaning that water saturation is extremely low and that there is no freely flowing water in the formation (Bruner and Smosna 2011). A number of other gas-bearing layers, such as the Rhinestreet Shale, overlie the Marcellus, and these layers should serve as barriers to vertical flow due to low permeability caused by low water saturation (in addition to other factors discussed in this section). Low permeability strata are also present above other black shales (Sandberg 1962; Kiteley 1978; Swezey 2008), thereby similarly restricting vertical flow in other sedimentary basins.

Cementation (both detrital and diagenetic) is another important process that reduces permeability. Both types of cementation reduce permeability, although diagenetic cement has generally a larger effect (e.g., quartz, calcite precipitation; Panda and Lake 1995). The greatest permeability reduction (often by several orders of magnitude; Archie 1950; Foster 1981 as cited in Bethke 1986; Panda and Lake 1995) is associated with the pore-bridging effect, where cement growth may block pores (Neasham 1977; Panda and Lake 1995). In addition to blocking flow through pore spaces, cement can also block flow through fractures. Cement-filled fractures are a common occurrence in sedimentary basins and can reduce the potential for preferential migration along these pathways (Gale and Holder 2010).

Overall, the preponderance of fine-grained rocks (i.e., shale, siltstone, and mudstone) and the layered structure of sedimentary basins will constrain the vertical permeability of bedrock above black shales toward the low end of measured values. Low permeability layers at depth in sedimentary basins are common, due to the effects of effective stress, cementation, and partial saturation. Only a thin low-*k* layer is needed to constrain vertical permeability to a low value, however, there are typically many low-*k* layers present, as are found above the Marcellus, Bakken, and other black shales (Figure 2; Sandberg 1962; Kiteley 1978; Swezey 2008; Ryder et al. 2012). Therefore, it is the rule rather than the exception that vertical permeability in the portions of these basins targeted for oil and gas development is comparable to that of low permeability shales/siltstones/mudstones rather than higher permeability types of rock.

## Conditions for Upward Flow

The necessary ingredient for upward flow is, of course, an upward head gradient. There are areas in sedimentary basins in which natural conditions create upward head gradients, and these are generated by one of two mechanisms—topography or overpressure. Topographic gradients create focused discharge areas (e.g., near a river valley or coast), whereas overpressured zones may be more wide spread but are inherently associated with low-*k* rocks (i.e., low upward fluxes). Under either driving mechanism, in order for upward flow to occur, the head gradient must be large enough to overcome density gradients associated with increasing salinity with depth. There are certainly instances of upward head gradients in the vicinity of some black shales, however, high upward head gradients would need to be sustained over thick sequences (typically >1000 m) of highly permeable bedrock to drive a significant amount of brine into shallow fresh groundwater. As discussed below, these two conditions are mutually exclusive, suggesting that high upward fluxes of brine are not physically plausible.

### Mechanisms That Can Generate Long Term Upward Head Gradients

Topographic gradients can establish long flow paths that penetrate to great depths and traverse entire basins (Senger et al. 1987; Garven et al. 1993). Consequently, brine can be transported over long distances and may eventually be able to migrate upward and mix with shallow fresh water. In general, however, shallow groundwater and surface water are fresh water (i.e., salinity is orders of magnitude lower than that of brine), indicating that large-scale upward brine fluxes are low relative to rates of precipitation-derived recharge.

More extensive areas of upward flow can be driven by overpressure, which often occurs in the deeper portions of many basins around the world (Hunt 1990). Overpressure arises from a disequilibrium state in which pore pressure's rate of generation exceeds its rate of dissipation. Pressure dissipation is primarily achieved by fluid flow out of the overpressured region and is therefore strongly influenced by permeability.

**Figure 4:**
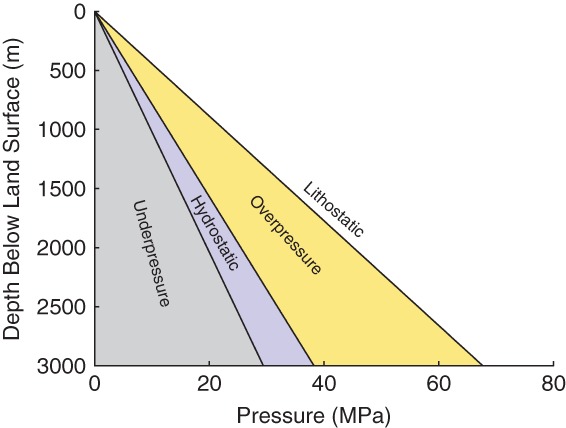
Pore pressure in overpressured, hydrostatic, and underpressured settings.

Two of the most important processes that can generate overpressure are disequilibrium compaction and the cracking of oil to natural gas (Swarbrick and Osborne 1998). Disequilibrium compaction most commonly occurs in basins undergoing rapid burial (e.g., the Gulf of Mexico; Dickinson 1956). As sediment accumulates, the increased overburden stress causes compaction and expulsion of pore water (Plumley 1980). If the permeability is too low to allow water to escape freely, then a portion of the increased stress will be borne by the trapped fluid and will result in overpressure (see pore pressure regimes in Figure 4). Other factors can also contribute to disequilibrium compaction, e.g., compressive tectonic stress (Berry 1973; Swarbrick and Osborne 1998) and cementation (Bethke 1986). Many basins in the United States are currently overpressured as a result of hydrocarbon generation (Law and Spencer 1998), which can be accompanied by large increases in fluid volume (especially the cracking of oil to gas). Estimates of the volume of gas generated per unit volume of oil cracked are approximately 550:1 (at STP), with pressures high enough to hydraulically fracture bedrock naturally being reached after only a few percent of oil has been cracked to gas (Barker 1990). For example, the Marcellus Shale was fractured by this mechanism, perhaps beginning as much as 300 million years ago, when the shale was deeply buried and within the temperature range conducive to rapid oil and gas generation (Engelder and Lash 2008).

Although overpressure is common in the deeper parts of many hydrocarbon-bearing basins, there are mechanisms that can result in underpressure, that is, pressures below hydrostatic that would induce downward rather than upward flow. One of these mechanisms is the extraction of hydrocarbons and resulting depressurization of the hydrocarbon-bearing formation (Swarbrick and Osborne 1998). Some examples of basins in the United States that are currently underpressured as a result of this process include the Frio Formation in the Gulf Coast Basin and the Woodbine Formation in the East Texas Basin (Kreitler 1989 and references therein). Underpressure can also occur naturally, primarily in gas-bearing basins that have been uplifted, cooled, and eroded (Hunt 1990; Corbet and Bethke 1992; Swarbrick and Osborne 1998). Some examples of natural underpressure include: the West Canada Basin, Alberta; Silurian Clinton sand, eastern Ohio; and Green River Basin, Wyoming (Swarbrick and Osborne 1998 and references therein).

### Vertical Head Gradient Limits

When upward flow occurs, there are two fundamental controls on the upward head gradient (d*h*/d*z*). The first is an upper limit imposed by the mechanical properties of rock, that is, if d*h*/d*z* is high enough it will fracture the rock and relieve built-up pressure. The second is a lower limit needed to overcome density stratification, due to the tendency for dense brine to form a stable fluid layer at depth, with less dense fresh water floating on top.

The upper limit to d*h*/d*z* is controlled by the maximum pore pressure that can be sustained without fracturing the rock. Rocks can be hydraulically fractured (either naturally or by humans) when pore pressure exceeds the least compressive stress, *σ*_min_, which holds fractures closed. The principal direction of *σ*_min_ varies with depth; it is typically vertical in shallow bedrock and horizontal at depth (Brown and Hoek 1978; Sheorey 1994). The vertical stress is the weight of overburden per unit area, meaning that a pore pressure that exceeds this value would physically push the overburden upward and create a fracture in the horizontal plane. When *σ*_min_ is horizontal (common at depth), fractures propagate vertically. In either case, the upper bound for *σ*_min_ is approximately the overburden stress (see Engelder 1993 for a full discussion of this topic), and therefore, the maximum fluid pressure that can be sustained without fracturing the rock is also approximately equal to the overburden stress. The magnitude of d*h*/d*z* under this limiting condition is, 
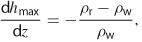
3 where *ρ*_r_ is the bulk density of overburden and *ρ*_w_ is the density of water (negative values of d*h*/d*z* indicate upward flow). With *ρ*_r_ at approximately 2300 kg m^−3^ and *ρ*_w_ at approximately 1230 kg m^−3^ for brine (assuming a salinity of 350,000 ppm at a temperature of 100 °C and 20 MPa pressure; Batzle and Wang 1992), Equation 3 indicates that the maximum upward head gradient is limited to about 1.

The lower limit to d*h*/d*z* is controlled by density gradients. Salinities in deep basin waters can range up to 400,000 ppm (Bassett and Bentley 1983; Hanor 1983), with densities up to 27% greater than fresh water (for a salinity of 400,000 ppm and the same temperature and pressure as before). Such density gradients are taken into account in basin-scale models of fluid flow by applying a correction factor to d*h*/d*z* in the fluid flow equations (Bethke 1989; Garven 1995). This correction factor is defined here as the brine density gradient (d*h*_b_/d*z*), 
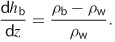
4

For *ρ*_b_ = 1230 kg m^−3^ and *ρ*_w_ = 1000 kg m^−3^, Equation 4 predicts a gradient of 0.23. Local density gradients will be much smaller, however, this estimate provides the head gradient needed to move a parcel of brine upward into an overlying fresh water aquifer (e.g., as envisioned by Myers [2012] and Rozell and Reaven [2012]). If density gradients are ignored, fluid flow models may incorrectly predict that flow is upward in areas where flow is actually downward (Senger and Fogg 1987).

### Head Gradient-Permeability Interdependence

The black shales targeted for HF are predominantly in basins where burial and rapid gas generation are no longer occurring. Consequently, in most cases where overpressure is present, it was likely generated in the past. Therefore, the question that must be answered is what permeabilities would allow overpressure to persist over the time since sediment deposition and rapid gas generation have ceased to be important. These timescales are generally on the order of tens to hundreds of millions of years for basins in the United States (Law and Spencer 1998).

In order for elevated pore pressure to persist over such long timescales, the permeability of overburden rocks must be sufficiently low to prevent pressure from diffusing across them. The magnitude of this permeability can be estimated from simple scaling relationships. For example, Deming (1994a) provided a solution to the one-dimensional (1D) groundwater flow equation that can be used to estimate permeability, 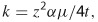
5 where *z* is overburden thickness, *α* is the compressibility of bedrock, *t* is the timescale for diffusion of pressure, and *μ* is the viscosity of water. Note that this relationship applies for homogeneous or heterogeneous rock, as well as for permeability dominated by fractures or the matrix. For timescales of 10 million to 100 million years, overburden thicknesses of 1000 to 5000 m (depth range of most black shales), *α* = 10^−9^ Pa^−1^ (a typical value for shale; Deming 1994a), and *μ* = 0.0005 Pa s, the permeability that would allow elevated pressure at these depths would be between 10^−23^ m^2^ and 10^−20^ m^2^. Note that this range of permeabilities is at the extreme low end of values reported in most standard groundwater hydrology texts (Freeze and Cherry 1979), but is consistent with the low permeability shales that are commonly found at depth (e.g., Kwon et al. 2001 and references therein; Corbet and Bethke 1992; Neuzil 1986). Such low permeabilities can be caused by a number of factors, as discussed previously.

There are clearly mechanisms that can drive upward flow in sedimentary basins, however, regardless of the driving mechanism, flow rates are low and flow paths are long. For topographically driven flow, water that penetrates deep enough to drive basin-scale brine migration must travel large horizontal distances (i.e., the length scale of a basin—typically tens to hundreds of kilometers) before reemerging at the surface. Over the past 200 million years, maximum cross basin (i.e., horizontal) flow rates at depth in many U.S. basins have been on the order of cm year^−1^ or lower (Garven et al. 1993; Garven 1995; Adams et al. 2004; Thornton and Wilson 2007), suggesting that cross basin, topographically driven flow of brine to the surface is associated with travel times of millions of years or longer. On the other hand, overpressured settings are inherently associated with very low permeability rock (e.g., 10^−23^ to 10^−20^ m^2^) and, therefore, upward fluid fluxes will be extremely small.

## Subsurface Changes Due to Hydraulic Fracturing and Hydrocarbon Production

One of the concerns regarding HF is whether the process could potentially cause brine or HF fluid to migrate upward to potable groundwater. Concerns are primarily related to the potential for induced fractures to increase vertical *k*_eff_ and transient elevated pressure to create upward head gradients. A large amount of monitoring data on fracture height growth has been recently published and shows that fractures have remained well below potable groundwater, as discussed in the next section. Conversely, there are no data on pressure propagation away from the fracture network, however, there are well known scaling relationships that can be readily used to bound the extent of pressure propagation. In any event, water introduced is most likely to be trapped by capillary tension, which causes gas-filled bedrock to soak up water like a dry sponge (Engelder 2012). These factors, discussed further below, suggest that HF fluid will be sequestered in the immediate vicinity of the fracture network.

### Physical Limits on Fracture Height Growth

Empirical data on vertical fracture growth (i.e., height above the target formation) have recently been published for the Barnett, Eagle Ford, Marcellus, Woodford, and Niobrara Shales (Fisher and Warpinski 2011; Davies et al. 2012). These data show the maximum height of fracture growth during each recorded stimulation and therefore, are indicative of the upper limit of fracture growth—generally on the order of 100 m. The tallest fractures tended to occur in the Marcellus Shale, where the median of the maximum fracture height distribution is a little more than 100 m, and the maximum recorded height is a little more than 500 m (Davies et al. 2012). These observations were made where depths of the Marcellus were approximately 1500 to 2500 m below land surface (Fisher and Warpinski 2011). Thus, for the tallest fractures, 1000 to 2000 m of intact bedrock remained above the upper edge of the fracture zone. The presence of a thick (>1000 m) bedrock interval above the top of the fracture zone was consistently found for all of the formations studied. Fisher and Warpinski (2011) provided a scaling analysis of the volume of fluid needed to hold fracture networks open and found that there was only enough HF fluid to propagate fractures upward on the order of 100 m above the target black shales, consistent with observations. They also noted that the tallest fractures observed to date were associated with growth up faults, however, even in these instances, the fracture heights were on the order of 100 m. The observations and scaling analysis presented by Fisher and Warpinski (2011) suggest that there is not enough HF fluid to propagate fractures upward across the thick bedrock intervals between deep black shales and shallow aquifers, contrary to speculation by others (Myers 2012; Rozell and Reaven 2012).

### Physical Limits on Pressure Propagation

Potential pressure propagation and displacement of natural formation brines have also been raised as HF-related concerns. Beyond the fracture network (i.e., just beyond the fracture face or at the outermost limits of fracture propagation), changes in pore pressure depend on rock and fluid properties that control pressure propagation. Equation 5 can be rearranged to solve for the distance (*s*) from the fracture network at which a change in pore pressure would occur in response to HF, yielding, 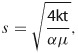
6 where all variables are the same as previously defined. For typical HF durations of 1–2 h, *k*_eff_ of 10^−20^ to 10^−16^ m^2^ (typical values for shale; Freeze and Cherry 1979), *α* = 10^−9^ Pa^−1^, and *μ* = 0.0005 Pa s at 50 °C, *s* ranges from 0.017 to 2.4 m. Thus, beyond the fracture network, the pressure disturbance in bedrock pore spaces is likely to be localized to the immediate vicinity of the fractures. Obviously, pressure propagation length scales would be much greater for more permeable rocks such as sandstone (as suggested by Myers 2012), but the dominant rock type in sedimentary basins is shale. In these lower permeability rocks, large-scale pressure propagation is unrealistic.

The short duration and localized pressure pulse associated with HF stimulations is in sharp contrast with the long duration and large-scale depressurization brought about by hydrocarbon production. For example, Equation 6 predicts that pumping from a gas well for 10 years would cause a pressure disturbance 5 to 500 m from the edge of the fracture network. Large-scale depressurization has been observed in oil and gas reservoirs, for example, in the Frio and Woodbine formations in Texas (Kreitler et al. 1987 as cited in Kreitler 1989). In the Palo Duro Basin, one analysis suggests that it would take approximately 10,000 years before pressures would recover to 90% of preproduction levels (Senger et al. 1987). In formations where hydrocarbon production has caused large-scale depressurization (e.g., the Frio formation), it is not known how long it might take for such an expansive area to return to preproduction pressures (Kreitler 1989). The scaling analysis (Equation 6) and these examples suggest that the HF pressure pulse is short lived and localized. Moreover, hydrocarbon production (i.e., pumping) will cause fluids to flow toward the fracture network over the long term, even after hydrocarbon production has ceased, thereby eliminating any short-term localized pressure effects of HF.

## Fluid Flow and Chemical Transport Evaluations

Modeling fluid flow is unfortunately complex at the depths where black shales occur, but is nevertheless necessary for modeling chemical transport. Some complicating issues arise in modeling analyses, such as the limited amount of data for these deep formations (Garven 1995), the potential for very low permeability layers, variations in temperature with depth, and the presence of variable salinity (and therefore density) and other fluid phases (e.g., oil and natural gas). The lack of such data is a potential limitation and additional data would certainly help in corroborating our understanding of deep fluid migration and the constraints on vertical flow.

The difficulties associated with flow modeling are not insurmountable, as long as they are dealt with in a physically sound manner. For example, the flow of oil and gas may not need to be specifically modeled if the flow of water is of greater interest; it may be reasonable in some instances to model hydrocarbon-bearing formations with an appropriately reduced permeability due to the effects of partial saturation. Modeling frameworks have been developed to handle variable density flow and heat transport (Langevin and Guo 2006; Pruess et al. 2011), both of which are important for simulating the coupled flow of fresh water and brine across thousands of meters of Earth's crust.

The key issue that is difficult to overcome in any case is the lack of data on deep formations. Such data limitations are always a problem, and therefore, model assumptions and results should always be evaluated with simple scaling analyses to provide reality checks. We have provided some scaling relationships in this article that may be helpful in some circumstances. In the following subsections, we discuss some simple approaches for bounding the magnitude of potential upward fluid fluxes and travel times—both of which are topics discussed in recent studies (Myers 2012; Rozell and Reaven 2012; Warner et al. 2012) that have been criticized or rebutted by others (Engelder 2012; Saiers and Barth 2012; Cohen et al. 2013).

### Magnitude of Upward Fluid Fluxes

Ultimately, one of the most critical questions to answer is the potential magnitude of the upper bound fluxes of brine and HF fluid. At a large scale, upward fluxes can only be a small fraction of regional recharge in areas where fresh groundwater occurs, otherwise the water would be saline. Throughout the United States, long-term average recharge is typically on the order of 0.001 to 1 m year^−1^ (Wolock 2003), however, there are few published estimates of upward brine and HF fluid fluxes. One recent study has proposed that natural (i.e., pre-HF) upward brine fluxes in the Appalachian Basin are 0.0031 to 6.7 m year^−1^ and that upward HF fluid fluxes would be even higher (Myers 2012). Such high upward fluxes (on the same order as total recharge) would cause shallow groundwater to be saline, contrary to observations of fresh groundwater found in sedimentary basins throughout the country (Eckhardt and Sloto 2012). In contrast, others have estimated that upward fluxes are many orders of magnitude lower (Jorgensen et al. 1996) or possibly zero, depending on permeability distributions and salinity gradients (Senger and Fogg 1987). Although the climate and basin settings vary in these cases, we think that estimates of upward flux presented by (Jorgensen et al. 1996) and Senger and Fogg (1987) (i.e., orders of magnitude lower than natural recharge or possibly zero) are more realistic and consistent with current knowledge of basin hydrology.

In general, scenarios that might cause a large upward flux of brine or HF fluid as a result of hydraulic fracturing are hard to conceive. As always, it would be good to have groundwater monitoring data to evaluate the extent of upward flow (or lack thereof), however, it is also difficult to conceive of a monitoring program that would provide meaningful information, given the constraints on pressure wave propagation and fluid flow at depth. For example, low permeability rocks prevent pressure waves from propagating in a short enough period to be observed over human timescales (Toth and Millar 1983), and a potential solute pulse would lag behind the pressure wave. In the absence of such data, there are compelling arguments based on geophysical monitoring and our physical understanding of the processes controlling fluid motion at depth suggesting that vertical fluxes associated with HF will be very small. For example, induced fractures are contained at depth (i.e., no direct hydraulic connection to shallow groundwater; Fisher and Warpinski 2011) and the HF pressure pulse is of too short a duration to affect natural hydraulic gradients. The effective hydraulic isolation of these formations is clearly demonstrated by the fact that fluids have been trapped at depth for tens to hundreds of millions of years. In fact, low permeability shales have been extensively evaluated as potential long-term storage sites for radioactive material due to their ability to trap fluids over geologic time (Horseman et al. 1996). Furthermore, oil and gas production following an HF stimulation will create a low pressure zone that will draw fluids downward rather than upward. Overall, there are not likely to be significant upward fluxes of brine or HF fluid to shallow groundwater, either before, during, or after an HF stimulation.

### Timescales for Chemical Transport

A variable related to the upward flux is the timescale for transport. The timescales for vertical transport, *t* = *Ln*/*q*, are constrained by physically plausible combinations of upward head gradients, permeability, effective porosity (*n*), and the depth interval between the fracture zone and shallow groundwater (*L*). For most basins in the United States that are targeted for HF, upward head gradients, if present, are likely to be relicts of past overpressure generating mechanisms (Law and Spencer 1998) and associated with low permeability rock (e.g., 10^−23^ m^2^ in some cases, as calculated previously). With this permeability, a unit head gradient (i.e., the maximum possible), and effective porosity values ranging from 10^−2^ (for the matrix) to 10^−5^ (for sparse fractures at high in situ stress; e.g., Gordon 1986; Liu et al. 1999), the travel time would be on the order of 10^5^ to 10^8^ year across a 100-m thick layer, i.e., very slow cross bedding flow. It is more likely, even in highly overpressured settings, that lateral flow through higher permeability strata toward the basin margins would be the dominant fluid migration route at depth (Deming 1994b), although this lateral flow is itself associated with long travel times (e.g., millions of years or longer; Kreitler 1989; Stueber and Walter 1991; Thornton and Wilson 2007). Short travel times (e.g., on the order of 10^1^ year) are not physically possible over thick sedimentary intervals above black shales.

## Summary

Much of the groundwork for understanding and modeling fluid flow and chemical transport within sedimentary basins has been previously established. Bulk limits and scaling functions are already available to provide reality checks on flow and transport analyses, some of which we have summarized in this paper. The characteristics of sedimentary basins in which black shales are located do not allow for rapid upward migration of HF fluid or brine over short timescales for the following reasons:

- Vertical permeabilities are dominated by the least permeable layer. The stratigraphy above black shales is typically dominated by shales, siltstones, and mudstones, and many of these layers have inherently low permeability, which is further reduced by high effective stress at depth, cementation, and partial saturation.
- Hydraulic fracturing affects a much smaller thickness of rock than that of the overburden. Similarly, the elevated pressures associated with HF are both short lived and localized to the fracture network, due to bedrock properties that limit pressure propagation at depth. Therefore, upward migration of HF fluid or brine would be controlled by natural vertical head gradients and would have to traverse a thick interval of low permeability bedrock in order to reach shallow groundwater.
- Natural upward head gradients do occur, and are driven by either topography or relict overpressure at depth. In either case, flow rates are low and timescales for transport are long (often >10^6^ years). In older overpressured basins, permeabilities required to maintain elevated subsurface pressure over geologic time are on the order of 10^−20^ m^2^ or lower, resulting in negligible vertical flow rates.

Our analysis and literature review indicate that where upward flow occurs, both permeability and flow rates are low, and therefore, timescales for transport are long. Overall, the rapid upward migration scenarios that have been recently suggested (Rozell and Reaven 2012; Myers 2012; Warner et al. 2012) are not physically plausible.

## References

[1] Adams JJ, Rostron BJ, Mendoza CA (2004). Coupled fluid flow, heat and mass transport, and erosion in the Alberta basin: Implications for the origin of the Athabasca oil sands. Canadian Journal of Earth Sciences.

[2] Archie GE (1950). Introduction to petrophysics of reservoir rock. AAPG Bulletin.

[3] Baird JK, Dyman TS (1993).

[4] Barker C (1990). Calculated volume and pressure changes during the thermal cracking of oil to gas in reservoirs. AAPG Bulletin.

[5] Bassett RL, Bentley ME (1983).

[6] Batzle M, Wang Z (1992). Seismic properties of pore fluids. Geophysics.

[7] Berry FAF (1973). High fluid potentials in California coast ranges and their tectonic significance. AAPG Bulletin.

[8] Bethke CM (1986). Inverse hydrologic analysis of the distribution and origin of Gulf Coast-type geopressured zones. Journal of Geophysical Research.

[9] Bethke CM (1989). Modeling subsurface flow in sedimentary basins. Geologische Rundschau.

[10] Bohne K, Nitsche C, van Genuchten MTh, Leij FJ, Leij FJ (1992). Requirements and use of indirect methods for estimating the hydraulic functions of unsaturated soils. Indirect Methods for Estimating the Hydraulic Properties of Unsaturated Soils Proceedings of the International Workshop.

[11] Bredehoeft JD (2003). Hydrodynamics of sedimentary basins. Encyclopedia of Physical Science and Technology.

[12] Brooks RJ, Corey AT (1964). Hydraulic properties of porous media.

[13] Brown ET, Hoek E (1978). Trends in relationships between measured in-situ stresses and depth. International Journal of Rock Mechanics and Mining Sciences.

[14] Bruner KR, Smosna R (2011).

[15] Clennell MB, Dewhurst DN, Brown KM, Aplin AC, Fleet AJ, Westbrook GK (1999). Permeability anisotropy of consolidated clays. Muds and Mudstones: Physical and Fluid-Flow Properties.

[16] Cohen HA, Parratt T, Andrews CB (2013).

[17] Connolly CA, Walter LM, Baadsgaard H, Longstaffe FJ (1990a). Origin and evolution of formation waters, Alberta Basin, Western Canada Sedimentary Basin. II. Isotope systematics and water mixing. Applied Geochemistry.

[18] Connolly CA, Walter LM, Baadsgaard H, Longstaffe FJ (1990b). Origin and evolution of formation waters, Alberta Basin, Western Canada sedimentary Basin. I. Chemistry. Applied Geochemistry.

[19] Corbet TF, Bethke CM (1992). Disequilibrium fluid pressures and groundwater flow in the Western Canada sedimentary basin. Journal of Geophysical Research.

[20] Davies RJ, Mathias S, Moss J, Hustoft S, Newport L (2012). Hydraulic fractures: How far can they go?. Marine and Petroleum Geology.

[21] Deming D (1994a). Factors necessary to define a pressure seal. AAPG Bulletin.

[22] Deming D (1994b). Fluid flow and heat transport in the upper continental crust.

[23] Desbarats AJ (1987). Numerical estimation of effective hydraulic conductivity in sand-shale formations. Water Resources Research.

[24] Dickinson G (1956). Geological aspects of abnormal reservoir pressures in Gulf Coast, Louisiana. AAPG Bulletin.

[25] Eckhardt DAV, Sloto RA (2012).

[26] Engelder T (2012). Capillary tension and imbibition sequester frack fluid in Marcellus gas shale (Letter). Proceedings of the National Academy of Sciences USA.

[27] Engelder T (1993). Stress regimes in the lithosphere.

[28] Engelder T, Lash GG (2008).

[29] Fisher K, Warpinski N (2011).

[30] Freeze RA, Cherry JA (1979). Groundwater.

[31] Freeze RA, Witherspoon PA (1967). Theoretical analysis of regional groundwater flow: 2. Effect of water-table configuration and subsurface permeability variation. Water Resources Research.

[32] Gale JFW, Holder JK (2010). Natural fractures in some US shales and their importance for gas production. Petroleum Geology Conference.

[33] Garven G (1995). Continental-scale groundwater flow and geologic processes. Annual Review of Earth and Planetary Sciences.

[34] Garven G, Ge S, Person MA, Sverjensky DA (1993). Genesis of stratabound ore deposits in the midcontinent basins of North America. 1. The role of regional groundwater flow. American Journal of Science.

[35] van Genuchten MT (1980). A closed-form equation for predicting the hydraulic conductivity of unsaturated soils. Soil Science Society of America Journal.

[36] Gordon MJ (1986). Dependence of effective porosity on fracture continuity in fractured media. Ground Water.

[37] Hanor JS (1983). Fifty years of development of thought on the origin and evolution of subsurface sedimentary brines. Revolution in the Earth Sciences: Advances in the Past Half-Century.

[38] Hogan JF, Mills SK, Hendrickx JMH, Ruiz J, Chesley JT, Asmerom Y (2007). Geologic origins of salinization in a semi-arid river: The role of sedimentary basin brines. Geology.

[39] Horseman ST, Higgo JJW, Alexander J, Harrington JF (1996).

[40] Hunt JM (1990). Generation and migration of petroleum from abnormally pressured fluid compartments. AAPG Bulletin.

[41] Jorgensen DG, Helgesen JO, Signor DC, Leonard RB, Imes JL, Christenson SC (1996).

[42] Kiteley LW (1978). http://pubs.er.usgs.gov/publication/oc78.

[43] Kreitler CW (1989). Hydrogeology of sedimentary basins. Journal of Hydrology.

[44] Kwon O, Kronenberg AK, Gangi AF, Johnson B (2001). Permeability of Wilcox shale and its effective pressure law. Journal of Geophysical Research.

[45] Langevin CD, Guo W (2006). MODFLOW/MT3DMS-based simulation of variable-density ground water flow and transport. Ground Water.

[46] Law BE, Law BE, Ulmishek GF, Spencer CW (1998). Abnormal pressure in hydrocarbon environments. AAPG Memoir 70. Abnormal Pressures in Hydrocarbon Environments.

[47] Liu J, Elsworth D, Brady BH (1999). Linking stress-dependent effective porosity and hydraulic conductivity fields to RMR. International Journal of Rock Mechanics and Mining Sciences.

[48] Miall AD (2008). The Sedimentary Basins of the United States and Canada.

[49] Morel-Seytoux HJ, Meyer PD, Nachabe M, Touma J, van Genuchten MT, Lenhard RJ (1996). Parameter equivalence for the Brooks-Corey and van Genuchten soils characteristics: Preserving the effective capillary drive. Water Resources Research.

[50] Myers T (2012). Potential contaminant pathways from hydraulically fractured shale to aquifers. Ground Water.

[51] Neasham JW (1977).

[52] Neuzil CE (1986). Groundwater flow in low-permeability environments. Water Resources Research.

[53] Nur A, Bredehoeft J, Walder J (1990). Time-dependent hydraulics of the Earth's crust. Role of Fluids in Crustal Processes.

[54] Pallatt N, Thornley D (1990). The role of bound water and capillary water in the evaluation of porosity in reservoir rocks. Advances in Core Evaluation: Accuracy and Precision in Reserves Estimation.

[55] Panda MN, Lake LW (1995). A physical model of cementation and its effects on single-phase permeability. AAPG Bulletin.

[56] Plumley WJ (1980). Abnormally high fluid pressure: Survey of some basic principles. AAPG Bulletin.

[57] Pruess K, Oldenburg C, Moridis G (2011).

[58] Rowan EL (2006).

[59] Rozell DJ, Reaven SJ (2012). Water pollution risk associated with natural gas extraction from the Marcellus Shale. Risk Analysis.

[60] Ryder RT, Trippi MH, Swezey CS, Crangle RD, Hope RS, Rowan EL, Lentz EE (2012).

[61] Ryder RT, Crangle RD, Trippi MH, Swezey CS, Lentz EE, Rowan EL, Hope RS (2009).

[62] Ryder RT, Swezey CS, Crangle RD, Trippi MH (2008).

[63] Sandberg CA (1962).

[64] Saiers JE, Barth E (2012). Potential contaminant pathways from hydraulically fractured shale aquifers, by T. Myers (Comment). Ground Water.

[65] Senger RK, Fogg GE (1987). Regional underpressuring in deep brine aquifers, Palo Duro Basin, Texas. 1. Effects of hydrostratigraphy and topography. Water Resources Research.

[66] Senger RK, Fogg GE, Kreitler CW (1987).

[67] Sheorey PR (1994). A theory for in situ stresses in isotropic and transverseley isotropic rock. International Journal of Rock Mechanics and Mining Sciences.

[68] Stueber AM, Walter LM (1991). Origin and chemical evolution of formation waters from Silurian-Devonian strata in the Illinois basin, USA. Geochimica et Cosmochimica Acta.

[69] Swarbrick RE, Law BE, Ulmishek GF, Osborne MJ (1998). Mechanisms that generate abnormal pressures: An overview. AAPG Memoir 70. Abnormal Pressures in Hydrocarbon Environments.

[70] Swezey CS (2009).

[71] Swezey CS (2008).

[72] Thornton MM, Wilson AM (2007). Topography-driven flow versus buoyancy-driven flow in the U.S. midcontinent: Implications for the residence time of brines. Geofluids.

[73] Toth J (1963). A theoretical analysis of groundwater flow in small drainage basins. Journal of Geophysical Research.

[74] Toth J (1962). A theory of groundwater motion in small drainage basins in central Alberta, Canada. Journal of Geophysical Research.

[75] Toth J, Millar RF (1983). Possible effects of erosional changes of the topographic relief on pore pressures at depth. Water Resources Research.

[76] Warner NR, Jackson RB, Darrah TH, Osborn SG, Down A, Zhao K, White A, Vengosh A (2012). Geochemical evidence for possible natural migration of Marcellus Formation brine to shallow aquifers in Pennsylvania. Proceedings of the National Academy of Sciences USA.

[77] Wolock DM (2003).

